# Racial Differences in Secondary Hyperparathyroidism

**DOI:** 10.5812/numonthly.9449

**Published:** 2013-08-13

**Authors:** Akihiko Kato

**Affiliations:** 1 Blood Purification Unit, Hamamatsu University Hospital, Hamamatsu, Japan

**Keywords:** Renal Dialysis, Parathyroid Hormone

***Dear Editor,***

I have recently read an interesting paper by Dr. Seck who had examined the prevalence of CKD-MBD in black African (Senegalese) patients on regular hemodialysis (HD) in a cross-sectional fashion (Nephrol-Urol Mon. 2012; 4(4): 613-616) (1). They showed that 57 out of the 79 patients complicated with CKD-MBD (72%) had a high turnover bone disease with a mean level of 984 pg/mL of intact parathyroid hormone (iPTH). Because mean calcium and phosphorous levels were not elevated (8.6 and 4.85 mg/dL), this marked increment of iPTH may be related to racial differences in the regulation of vitamin D-PTH axis.

Autopsy studies have demonstrated that parathyroid mass is increased in blacks compared with whites (2). There is a 4.4-fold higher risk for severe secondary hyperparathyroidism (iPTH > 500 pg/mL) in black patients than in white patients at dialysis initiation (3). African-American HD patients have iPTH levels that are higher than expected in relation to bone histology (4). Blacks with advanced CKD not yet on dialysis also have lower 25(OH)D and higher iPTH concentrations with declining kidney function compared with whites, independent of FGF-23 concentrations (5). So, there may be a unique mechanism by which blacks develop secondary hyperparathyroidism, such as skeletal resistance to PTH, or more activation of calcium-sensing receptor in the parathyroid gland.

Although current guidelines on the management of CKD-MBD recommend screening and treating abnormalities in mineral metabolism, none of them take into account for racial differences. Thus, further evaluation will be needed to realize whether current guidelines are truly adequate for all races/ethnicities. 
